# Apolipoprotein-J prevents angiotensin II-induced apoptosis in neonatal rat ventricular cells

**DOI:** 10.1186/s12944-015-0118-y

**Published:** 2015-09-21

**Authors:** Yanzhuo Ma, Lingfeng Kong, Kai Nan, Shuying Qi, Leisheng Ru, Chao Ding, Dongmei Wang

**Affiliations:** Department of Cardiology, Bethune International Peace Hospital, Shijiazhuang, Hebei China; Health and Medical Development Research Center of Hebei Province, Shijiazhuang, Hebei China

**Keywords:** AngII, ApoJ, Apoptosis, Neonatal rat ventricular cells

## Abstract

**Background:**

Up-regulation of angiotensin II (AngII) occurs in cardiac diseases, such as congestive heart failure, cardiac hypertrophy, myocardial ischemia and atrial fibrillation, which represent major health problems. Evidence from *in vivo* studies suggests that the level of Apolipoprotein-J (ApoJ) is also elevated but plays a protective role in cardiovascular disease. This study aimed to evaluate the protective effects of ApoJ against cytotoxicity of AngII in neonatal rat ventricular cells (NRVCs).

**Methods and results:**

In culture, NRVCs were damaged by exposure to AngII, and ApoJ overexpression using an adenovirus vector significantly reduced the AngII-induced cell injury. ApoJ also prevented AngII from augmenting Nox2/gp91^phox^ expression. The reactive oxygen species (ROS) scavenger, Mn(III)TBAP, showed similar results of attenuating AngII-induced cell damage. Furthermore, ApoJ overexpression increased phosphorylation of Akt, and the phosphatidylinositol 3-kinase (PI3K) inhibitor LY294002 diminished the antioxidant effects of ApoJ, and prevented the protective effect of ApoJ against the cytotoxicity of AngII. Moreover, upregulation of nuclear factor κB (NF-κB) p65 expression and phosphorylation of p38 mitogen-activated protein kinase (MAPK) mediated by AngII in cultured NRVCs were significantly inhibited by overexpression of ApoJ. The p38 MAPK inhibitor SB203580 and the NF-κB inhibitor PDTC protected NRVCs from injury caused by AngII.

**Conclusions:**

ApoJ serves as a cytoprotective protein in NRVCs against cytotoxicity of AngII through the PI3K-Akt-ROS and MAPK/ NF-κB pathways.

## Background

Apolipoprotein-J (ApoJ), also known as Clusterin, is a multifunctional glycoprotein widely present in tissues and body fluids. It has been implicated in such diverse processes as sperm maturation, regulation of complement activation, programmed cell death, tissue remodeling and lipid transport. Expression of ApoJ is upregulated in acute myocardial infarction, atherosclerosis, myocarditis, oxidative stress, inflammation and after injury in general. Klock *et al.* [1] demonstrated that apoptotic processes are responsible for increased ApoJ expression, and the function of ApoJ has been proposed to limit tissue injury and/or promote tissue remodeling [2].

The amyloid precursor protein (APP) is cleaved by β - and γ-secretase to release the amyloidogenic β-amyloid peptides (Aβ) and the APP intracellular domain (AICD). It has been believed that Aβ and AICD are involved in the onset and progression of Alzheimer’s disease (AD). AICD has been shown to potentiate endoplasmic reticulum (ER) stress-induced apoptosis, and knockdown of ApoJ mimicks the effect of AICD, suggesting that ApoJ exerts a prosurvival function against ER stress-mediated cell death [3]. Schwarz *et al.* [4] reported that ApoJ has cytoprotective and anti-inflammatory properties, by interacting with diverse substrates, and they demonstrated that ApoJ could inactivate C5b-9 complement complexes and reduce the cytotoxic effects of modified LDL on cells. ApoJ overexpression using an adenovirus vector was shown to inhibit vascular smooth muscle cells migration, adhesion and proliferation and to be beneficial to endothelial cells in injured blood vessels [5]. Blockage of secreted ApoJ by a monoclonal antibody resulted in increased apoptosis of neuroblastoma cells exposed to the chemotherapeutic drug doxorubicin, and its expression was required for resistance to apoptotic cell death induced by the chemotherapeutic drug doxorubicin [6]. In the heart, ApoJ was shown to exert protective effects on ischemically-challenged H9c2 cells and isolated adult ventricular rat cardiomyocytes [7]. In addition, ApoJ-deficient but not wild-type mice were found to exhibit impairment of cardiac function and severe myocardial scarring. In general, activation of ApoJ may play an important role in reducing apoptosis in normal and diseased cells.

Renin-angiotensin system (RAS) activation is known to contribute to increased angiotensin II (AngII) levels, thus leading to vascular damage, inflammation, oxidative stress and atherosclerosis [8–10]. This study aimed to determine whether ApoJ overexpression provides cardioprotection against AngII-induced injury and to explore the mechanisms by which ApoJ exerts its protective effects.

## Results

### ApoJ overexpression attenuates cell injury induced by AngII

**Fig. 1:**
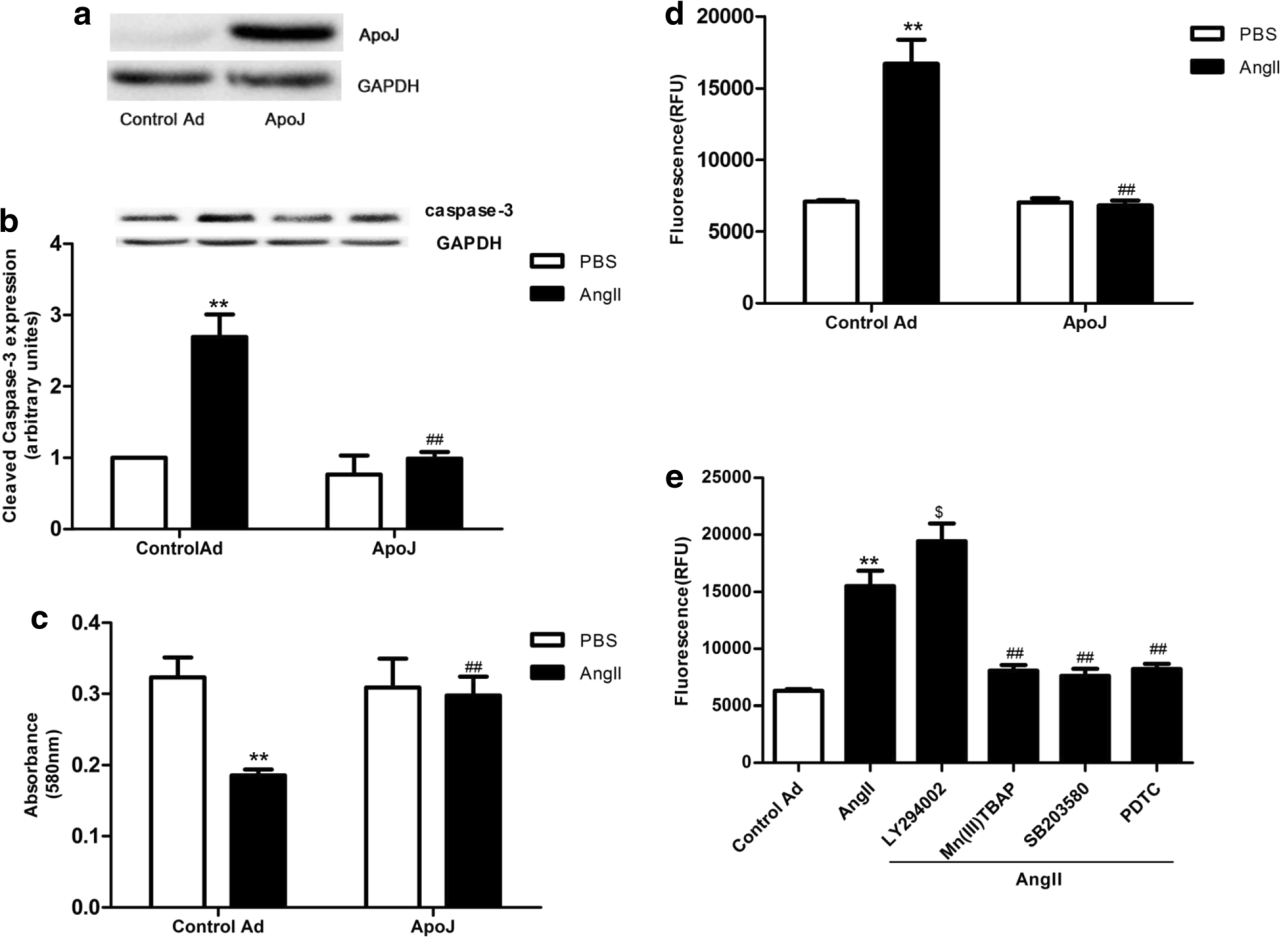
ApoJ overexpression by infection with recombinant adenovirus prevents AngII-induced apoptosis in NRVCs. **a** Apo J expression after infection by recombinant adenovirus was determined by Western blot (*n* = 3/group). **b** Cleavage of caspase-3 was stimulated by AngII and inhibited by ApoJ (*n* = 3/group). **c** Control and ApoJ-transduced NRVCs were incubated with AngII for 24 h, and then cell viability was determined by MTT assay (*n* = 8/group). **d** Apoptosis of cardiomyocytes was determined by caspase-3/7 activity assay. Apoptosis of control adenovirus infected NRVCs was significantly increased, while ApoJ-overexpressing NRVCs showed resistance to AngII-induced cell injury (*n* = 8/group). **e** LY294002, Mn(III)TBAP, SB203580 and PDTC were pretreated in NRVCs for 1 h, and then the cells were incubated with AngII for 24 h. Cell apoptosis was determined by caspase-3/7 activity assay (*n* = 8/group). **P < 0.01 versus control Ad/PBS group; ^##^ *P* < 0.01 versus control Ad/AngII group, ^$^ *P < 0.05* versus control Ad/AngII group

To determine whether ApoJ exerts beneficial effects on cardiomyocytes against AngII, ApoJ expression was achieved by infection with recombinant adenovirus in NRVCs. ApoJ expression was markedly increased in the Ad-ApoJ-infected NRVCs but not in control adenovirus-infected cells as confirmed by Western blot analysis with an anti-rat ApoJ antibody (Fig. 1a). We then determined the effect of ApoJ in AngII-induced cell injury, and 0.1 μM AngII was added into the adenovirus-infected NRVCs 36 h after infection. ApoJ overexpression conferred a significant resistance to cell death induced by AngII, compared with the control adenovirus-infected cells when exposed to AngII for 24 h. As shown in Fig. 1b, expression of cleaved caspase-3 was enhanced by AngII, and ApoJ overexpression markedly attenuated it. Similar results were observed in the MTT assay, which showed that AngII markedly decreased cell viability, and ApoJ overexpression significantly prevented the decrease in viability during exposure to AngII (Fig. 1c). Furthermore, the percentage of apoptotic cardiomyocytes which increased after AngII administration was markedly reduced by ApoJ overexpression (Fig. 1c). These results suggest that increased ApoJ expression plays a critical role in protecting cardiomyocytes from cell death induced by apoptotic agents. Next, we investigated which downstream effectors of AngII [phosphatidylinositol-3-kinase-Akt (PI3K/Akt), reactive oxygen species (ROS), mitogen-activated protein kinase (MAPK) p38 or nuclear factor kB (NF-kB) p65] mediate apoptosis and might be depressed by ApoJ to prevent AngII-induced apoptosis. We used a PI3K-Akt inhibitor [2-(4-morpholinyl)-8-phenyl-4 H-1-benzopyran-4-one, LY294002], ROS scavenger [Mn(III)TABP], MAPKp38 inhibitor (SB203580) or NF-κB p65 inhibitor (pyrrolidine dithiocarbamate, PDTC) in the presence of AngII. As shown in Fig. 1e, Mn(III)TBAP, SB203580 and PDTC significantly reduced the percent of apoptotic cells induced by AngII, while LY294002 further increased cell apoptosis. These results indicate that AngII mediates apoptosis through MAPK38, NF-kB, and ROS, and PI3K/Akt is a potential locus where ApoJ could act to ameliorate AngII-stimulated apoptosis.

### Phosphatidylinositol-3-kinase contributes to the anti-apoptotic effect of ApoJ

**Fig. 2:**
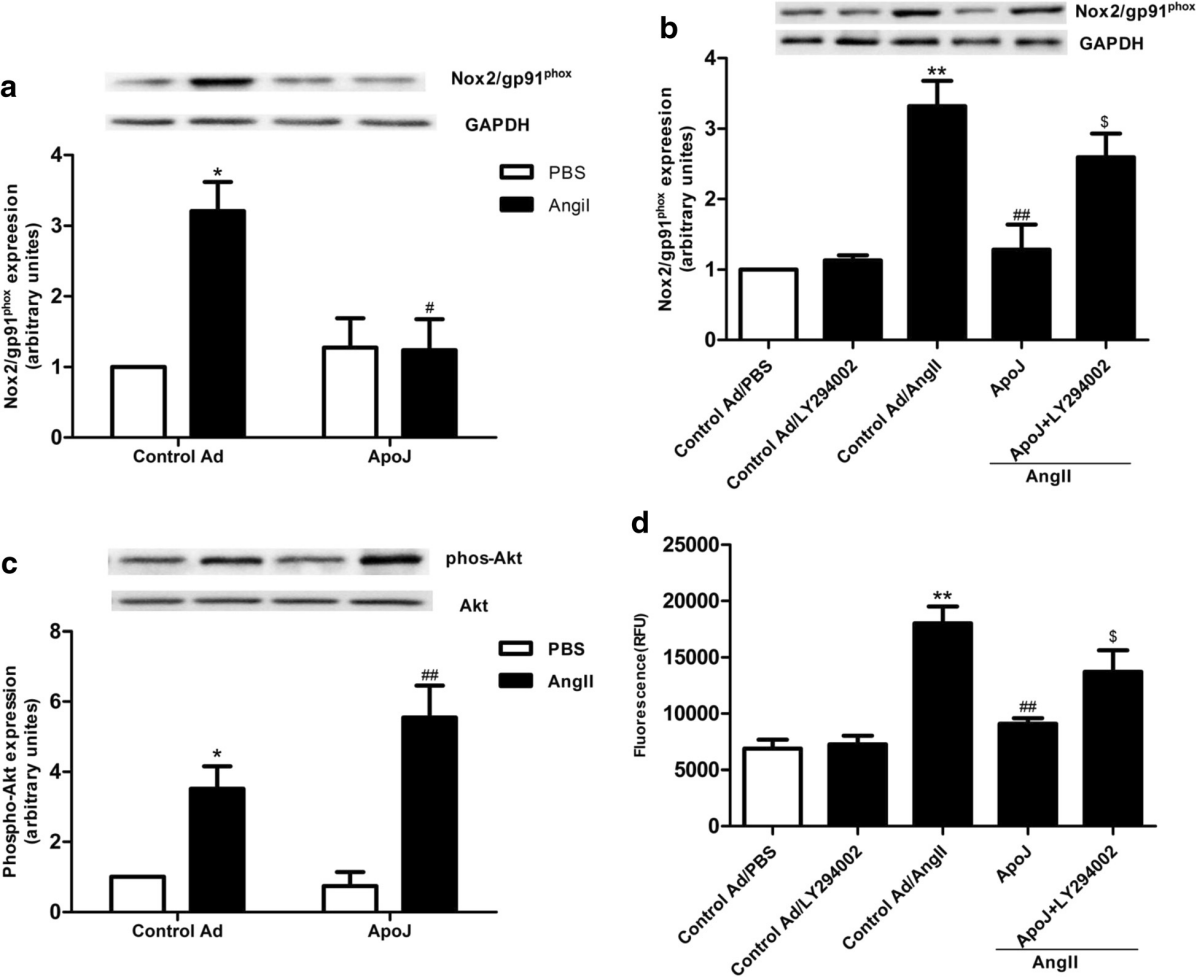
ApoJ attenuates the increased ROS production via PI3K-Akt pathway induced by AngII. **a** Western blot analysis of Nox2/gp91phox expression in NRVCs. Nox2/gp91phox expression was enhanced by AngII in control adenovirus-infected cells, while ApoJ-transduced cells inhibited its expression (*n* = 3/group). **b** LY294002 was used to pretreat ApoJ-transduced NRVCs for 1 h, and then the control and ApoJ-transduced NRVCs were incubated with AngII for 24 h. Nox2/gp91phox expression was determined by Western blot (*n* = 3/group). **c** AngII increased pAkt expression in control infected NRVCs, and ApoJ further augmented its expression. **d** Cell apoptosis caused by AngII increased in ApoJ-overexpressing cells treated with LY294002. ^*^ *P* < 0.05 versus control Ad/PBS group, ^**^ *P* < 0.01 versus control Ad/PBS group; ^#^ *P* < 0.05 versus control Ad/AngII group, ^##^ *P* < 0.01 versus control Ad/AngII group,^$^ *P* < 0.05 versus ApoJ group

In order to determine if ApoJ exerts protection on NRVCs by reducing ROS production, we tested Nox2/gp91phox expression. As shown in Fig. 2a, ApoJ overexpression markedly prevented AngII from stimulating the Nox2/gp91phox expression, as compared with the control adenovirus-infected cells when exposed to AngII for 24 h. To define this putative signaling pathway, we added the inhibitor of PI3K-Akt, LY294002, to NRVCs. When LY294002 was added to the ApoJ-overexpressing cells, it abrogated the effect of ApoJ on Nox2/gp91phox expression (Fig. 2b). These results emphasize the involvement of the PI3K-Akt pathway in the ApoJ-mediated inhibition of AngII-induced ROS production in NRVCs. Since the suppression of ROS production in response to ApoJ was found to depend on the PI3K-Akt pathway, we next inquired whether ApoJ treatment of NRVCs would result in elevated phosphorylation of Akt. As depicted in Fig. 2c, Akt phosphorylation increased after exposure to AngII, and ApoJ overexpression further significantly stimulated its expression. We also investigated if PI3K-Akt inhibitor (LY294002) affects the ability of ApoJ to protect NRVC from AngII-induced apopoptic injury. As shown in Fig. 2d, cell apoptosis caused by AngII was significantly decreased by ApoJ overexpression, while LY294002 significantly reduced the ability of ApoJ to protect against AngII-induced cell injury.

### ApoJ prevents MAPK and NF-κB activation

**Fig. 3:**
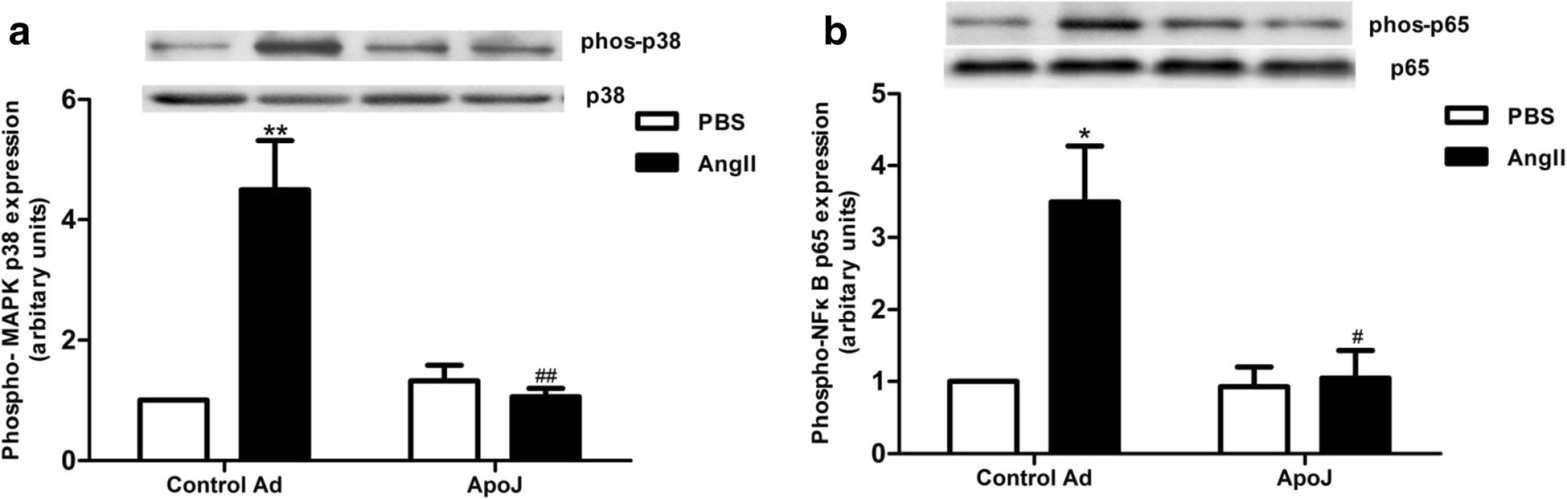
Effects of ApoJ on expression of p38 kinase and NF-κB p65 in NRVCs. **a** MAPK p38 expression in NRVCs. Phosphorylated MAPK p38 expression was enhanced by AngII in control infected cells, while ApoJ-transduced cells inhibited its expression (*n* = 3/group). **b** Western blot analysis of NF-κB expression in NRVCs. NRVCs were infected with control or ApoJ-expressing adenovirus for 36 h and then incubated with AngII for 24 h. Addition of AngII increased phosphorylated NF-κB expression, while ApoJ-transduced cells significantly decreased its expression (*n* = 3/group). *P < 0.05 versus control Ad/PBS group; **P < 0.01 versus control Ad/PBS group, ^#^ *P* < 0.05 versus control Ad/AngII group, ^##^ *P* < 0.01 versus control Ad/AngII group

To investigate whether the anti-apoptotic pathway employed by ApoJ involves MAPK p38 and NF-κB p65, we analyzed their expression levels. As shown in Fig. 3a, results of the Western blots demonstrated that AngII induced a significant increase of phosphorylated p38 protein, and this increase was abolished by ApoJ. Addition of exogenous AngII significantly increased NF-κB expression as compared to control adenovirus-infected cells, and the increased phosphorylation of NF-κB p65 was also significantly attenuated by ApoJ (Fig. 3b). These results indicated that ApoJ protects NRVCs from apoptosis via the MAPK/ NF-κB pathway initiated by AngII.

## Discussion

This study provides the first experimental evidence showing that ApoJ attenuates AngII-induced apoptosis in NRVCs. Furthermore, this study demonstrates that the cytoprotection of ApoJ against AngII may be mediated through activation of the PI3k-Akt pathway, thus suppressing ROS production, and through inhibiting the MAPK/NF-κB pathway.

ApoJ is associated with subclasses of plasma high density lipoproteins (HDL), and elevated levels of HDL have been inversely correlated with the risk of coronary heart disease, suggesting that the protective effect exerted by ApoJ is related to HDL. ApoJ is a circulating glycoprotein, which can be induced by injury, such as Alzheimer’s disease, atherosclerosis and myocardial infarction [11, 12]. In addition, ApoJ not only has been associated with apoptotic cell death but also demonstrated to be expressed in apoptosis-resistant cells [13] and required for cell survival [14–17].

Previous studies have reported that ApoJ participates in lipid transport, apoptosis regulation, protection of cellular membranes and promotion of cell-cell interactions [12]. The serum level of ApoJ level was found to be significantly increased in type II diabetes patients and in patients with either a developing coronary heart disease or myocardial infarction as compared to healthy individuals, but its level showed no correlation with the number of vessels with documented atherosclerotic damage [18, 19]. Given the significant elevated level of ApoJ that occurs in injury processes, an increased ApoJ level may be a strong indication of vascular damage. However, other studies have reported the decrease of ApoJ level after cardiac injury. For example, Cubedo *et al.* [20] observed that the serum ApoJ level was markedly decreased after the onset of acute myocardial infarction (AMI) compared with control patients and returned to control levels between 72 and 96 h after hospital admission. Considering the contradictory findings regarding ApoJ in cardiovascular disease, its benefit or detriment to the heart…….

In the last decade, multiple lines of evidence have suggested that ApoJ is associated with cell apoptosis. Poulakou *et al.* [21] discovered that serum ApoJ levels are positively associated with coronary artery disease. Methoxyacetic acid (MAA) treatment has been demonstrated to cause a localization of ApoJ to the cytoplasm, which precluded apoptotic cell death [22]. Overexpression of ApoJ was also shown to protect cells from apoptosis [15, 16], and blocking of ApoJ biosynthesis by antisense oligonucleotides caused an increase in cellular apoptosis *in vitro* [17, 23]. Bailey *et al.* [24] demonstrated that ApoJ could delay cell apoptosis in heat-stressed testes, while other studies using ApoJ knockout mice showed that ApoJ could limit the severity of induced auto-immune autocarditis [25] and exacerbate brain injury after neonatal hypoxia-ischemia [26]. ApoJ has been proposed to be an antioxidant agent capable of protecting cells from apoptosis induced by ROS [17]. Pathology studies have discovered that ApoJ is constitutively expressed in atrial but not in ventricular myocytes in healthy adult mouse and rat hearts, while myocardial injury markedly upregulates the level of ApoJ in ventricular myocytes. The current study aimed to determine if elevated ApoJ exerts protective effects in cardiomyocytes against the cytotoxicity of AngII. Therefore, we overexpressed ApoJ in NRVCs to define this function and found that it prevented AngII-induced cell damage by increasing cell viability and decreasing cell apoptosis. In addition, we also found that MAPKp38 inhibitor, NF-κB p65 inhibitor and ROS scavenger exerted effects on apoptosis of NRVCs similar to that afforded by ApoJ, while PI3K-Akt inhibitor worsened cell damage in the presence of AngII, indicating that PI3K-Akt, MAPKp38, NF-κB p65 and ROS may take part in the signals that regulate the response of ApoJ to AngII-induced cell damage.

Apoptosis is a complex process that occurs through many pathways [27]. ROS are well demonstrated as agents that cause cell apoptosis. AngII has been found to enhance NADPH oxidase activity that leads to increased ROS production, thus initiating a number of pathological processes. In this study, we found that adenovirus-mediated overexpression of ApoJ abrogated the elevated ROS production induced by AngII, as determined by reduced Nox2/gp91^phox^ expression in NRVCs. These results suggest that changes in the downstream ROS pathways may take part in the mechanisms of the anti-apoptotic effects of ApoJ.

AngII receptors are G-protein coupled receptors, AngII binds to AT1R to activate downstream signaling molecules such as PI3K, and activation of PI3K leads to Akt phosphorylation. The PI3K-Akt pathway is activated in response to various stimuli, growth factors and hormones. Once activated, Akt phosphorylates many cytosolic and nuclear substrates that are involved in numerous cellular responses, including promotion of cell survival, control of cell cycle progression and regulation of cell growth. ApoJ serves as a circulating glycoprotein, might bind to AT1R to facilitate Akt phosphorylation in NRVCs. In this study, we found that ApoJ overexpression markedly augmented pAkt expression. However, administration of the specific PI3K inhibitor LY294002 significantly impaired the ability of the ApoJ-expressing adenovirus from reducing AngII-stimulated expression of Nox2/gp91phox. Thus, ApoJ may exert its suppressive effects on Nox2/gp91^phox^ expression via activating the PI3K-Akt pathway.

Accumulating lines of evidence have demonstrated that up-regulation of NF-κB plays a critical role in physiological processes, including cell survival and proliferation, and in inflammatory diseases such as atherosclerosis [28]. NF-κB proteins are found in many types of cells and respond to various stimuli, such as cytokines and bacterial or viral antigens [29–31]. Previous studies have demonstrated that AngII could activate the NF-κB pathway. Here, we investigated the potential effect of ApoJ on NF-κB activation and related signal transduction in NRVCs treated with AngII. We found administration of AngII activated NF-κB p65 expression, and this NF-κB activation could be blocked by overexpression of ApoJ.

MAPK p38 is also responsive to various stimuli and is involved in regulation of cellular processes, such as proliferation, cell survival and apoptosis [32]. We investigated the potential effect of ApoJ on MAPK p38 activation in NRVCs treated with AngII, and we found administration of AngII activated MAPK p38 expression, and this MAPK p38 activation could also be blocked by overexpression of ApoJ.

## Conclusions

**Fig. 4:**
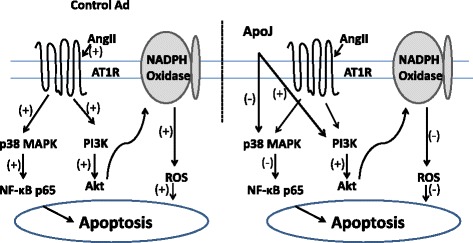
Signaling pathway diagram of ApoJ action in NRVCs. 1) In control adenovirus-infected cells, AngII stimulates phosphorylation of MAPK and Akt, which enables the activation of NF-κB and NADPH oxidase and consequently induction of apoptosis in NRVCs. 2) In NRVCs, p38 MAPK, NF-κB, Akt and NADPH oxidase activated by AngII are blocked by overexpression of ApoJ

In conclusion, this study demonstrated the ability of ApoJ to inhibit AngII-induced cell damage via activating PI3K activity, thus reducing ROS production, and via inhibition of the MAPK/NF-κB pathway (See Fig. 4 for an illustration of the signaling pathway of ApoJ action in NRVCs). Although these results may not be extrapolated directly to *in vivo* situations, this study provides evidence of a possible mechanism by which ApoJ exerts protection in NRVCs and may therefore provide a link to the prevention of atherosclerotic risk.

## Methods

### Animals

Neonatal Sprague–Dawley (SD) rats were obtained from the Department of Experimental Animals of Hebei University (Shijiazhuang, Hebei, China). The investigation was approved by the Animal Care and Use Committee at Bethune International Peace Hospital (Shijiazhuang, Hebei, China). The experiment was carried out in accordance with The Code of Ethics of the World Medical Association (Declaration of Helsinki).

### Myocyte isolation and cell culture

Single ventricular myocytes were obtained by enzymatic dissociation using collagenase (type II) and pancreatin as described elsewhere with some modifications. Ventricular myocardial tissue from neonatal SD rats (aged 1 d, Hebei Medical University) were homogenized and dissociated with collagenase II and pancreatin six times for 20 min each time. The first cell suspension was discarded, while the rest of the cell suspensions were dispensed with FBS and mixed together in 10-cm plates for 1.5 h, allowing enrichment for cardiomyocytes by differential adhesion. The supernatant was then plated onto a new dish with DMEM containing 10 % FBS at 37 °C.

#### Adenoviral infection of NRVCs

The ApoJ-expressing adenovirus was generated using pAD-ApoJ-IRES-EGFP adenoviral vector. Adenovirus generated from pAD-IRES-EGFP was used as control, the adenoviral vectors were synthesized using the Gateway Cloning System (invitrogen). Isolated NRVCs were infected with indicated adenoviruses at a multiplicity of infection (MOI) of 200 with serum-free DMEM. After 4 h, supernatants were removed and replaced with DMEM containing 10 % FBS. After 36 h, supernatants were removed, and NRVCs were given different treatments.

### 3-(4,5-Di methylthiazol-2-y l)-2,5-diphenylt etrazolium bromide (MTT) assay

Cell viability was estimated using the MTT assay (Sigma, St. Louis, MO, USA). Cells (2 × 10^4^ cells/ml) were cultured in complete medium for 24 h on 96-well plates. The medium on the cell cultures was replaced by fresh medium containing 0.5 % FBS, and then the cells were given different treatments. After the treatments, 20 μL of MTT at a concentration of 5 mg/ml was added, and the cells were incubated for 4 h. Thereafter, the medium was discarded, and 150 μl of DMSO was added for 10 min. The absorbance at 570 nm was recorded in each well using an ELISA microplate reader. All assays were performed in triplicate and repeated three times.

### Caspase-3/7 activity assay

Apo-ONE Homogeneous Caspase-3/7 Assay (Promega, Madison, WI, USA) was used to measure apoptosis in NRVCs. Briefly, cells plated in 96-well plates at a density of 10^4^ cells/well were given different treatments, and then 100 μl of Apo-ONE Homogeneous Caspase-3/7 Reagent was added to each well. The plate was incubated for 1 h at room temperature using a plate shaker at 350 rpm. Subsequently, fluorescence was measured in a fluorometer with excitation at 499 nm and emission at 521 nm. All assays were performed in triplicate and repeated three times.

### Immunoblotting

After being rinsed in cold PBS three times, cells were homogenized in RIPA buffer. The supernatant was then centrifuged at 120,000 × *g* for 15 min at 4 °C. Samples (10–20 mg) were run on SDS-PAGE gels, transferred to PVDF filter membranes and Western blotted with monoclonal antibodies against phosphor-Akt (Ser473) (Cell Signaling Technology, Beverly, MA, USA), Nox2/gp91^phox^ (Abcam, Cambridge, MA, USA), Phospho-NF-κB p65 (Ser536) (Cell Signaling Technology), Phospho-p38 MAPK (Thr180/Tyr182) (Cell Signaling Technology), cleaved caspase-3 (Asp175) (Cell Signaling Technology), ApoJ (EterLife, Birmingham, UK) and β-Actin (Sigma). PVDF membranes were then incubated with HRP-conjugated anti-rabbit immunoglobulin G antibody (Santa Cruz Biotechnology, Santa Cruz, CA, USA) for 1 h. The blot was developed with an ECL-Plus chemiluminescence reagent kit and visualized with the UVP Bio-Imaging System. Blot densities were analyzed using Image J software.

### Statistical analysis

All values in the text and figures are presented as the mean ± SD of n independent experiments. All data (except Western blot density) were subjected to ANOVA followed by Bonferroni correction for *post hoc* tests. Western blot densities were analyzed with the Kruskal-Wallis test followed by Dunn *post hoc* tests. *P* < 0.05 was considered statistically significant.

## Abbreviations

ApoJ: Apolipoprotein-J
AICD: APP intracellular domain
ER: Endoplasmic reticulum
AngII: Angiotensin II
ROS: Reactive oxygen species
PDTC: Pyrrolidine dithiocarbamate
PI3K-Akt: Phosphatidylinositol-3-kinase/Akt
LY294002: 2-(4-morpholinyl)-8-phenyl-4 H-1-benzopyran-4-one
MAPK: Mitogen-activated protein kinase
NF-κB: Nuclear factor κB
NRVCs: Neonatal rat ventricular cells
